# Premeiotic 24-nt phasiRNAs are present in the *Zea* genus and unique in biogenesis mechanism and molecular function

**DOI:** 10.1073/pnas.2402285121

**Published:** 2024-05-13

**Authors:** Junpeng Zhan, Sébastien Bélanger, Scott Lewis, Chong Teng, Madison McGregor, Aleksandra Beric, Michael A. Schon, Michael D. Nodine, Blake C. Meyers

**Affiliations:** ^a^National Key Laboratory of Crop Genetic Improvement, Huazhong Agricultural University, Wuhan 430070, China; ^b^Hubei Hongshan Laboratory, Wuhan 430070, China; ^c^Donald Danforth Plant Science Center, St. Louis, MO 63132; ^d^The James Hutton Institute, Dundee, Scotland DD2 5DA, United Kingdom; ^e^Division of Biology and Biomedical Sciences, Washington University, St. Louis, MO 63130; ^f^Genome Center, University of California, Davis, CA 95616; ^g^Department of Plant Sciences, University of California, Davis, CA 95616; ^h^Division of Plant Science and Technology, University of Missouri, Columbia, MO 65211; ^i^Laboratory of Molecular Biology, Wageningen University, Wageningen 6708 PB, the Netherlands

**Keywords:** maize, teosinte, phasiRNA, small RNA, nanoPARE

## Abstract

Reproductive phasiRNAs (phased, small interfering RNAs) are broadly present in angiosperms and play crucial roles in sustaining male fertility. While the premeiotic 21-nt (nucleotides) phasiRNAs and meiotic 24-nt phasiRNA pathways have been extensively studied in maize (*Zea mays*) and rice (*Oryza sativa*), a third putative category of reproductive phasiRNAs–named premeiotic 24-nt phasiRNAs–have recently been reported in barley (*Hordeum vulgare*) and wheat (*Triticum aestivum*). To determine whether premeiotic 24-nt phasiRNAs are also present in maize and related species and begin to characterize their biogenesis and function, we performed a comparative transcriptome and degradome analysis of premeiotic and meiotic anthers from five maize inbred lines and three teosinte species/subspecies. Our data indicate that a substantial subset of the 24-nt phasiRNA loci in maize and teosinte are already highly expressed at the premeiotic phase. The premeiotic 24-nt phasiRNAs are similar to meiotic 24-nt phasiRNAs in genomic origin and dependence on DCL5 (Dicer-like 5) for biogenesis, however, premeiotic 24-nt phasiRNAs are unique in that they are likely i) not triggered by microRNAs, ii) not loaded by AGO18 proteins, and iii) not capable of mediating *PHAS* precursor cleavage. In addition, we also observed a group of premeiotic 24-nt phasiRNAs in rice using previously published data. Together, our results indicate that the premeiotic 24-nt phasiRNAs constitute a unique class of reproductive phasiRNAs and are present more broadly in the grass family (Poaceae) than previously known.

## Significance

We previously reported two classes of reproductive phasiRNAs (phased, small interfering RNAs) in maize, the premeiotic 21-nt (nucleotides) phasiRNAs and the meiotic 24-nt phasiRNAs. Here, we report a third class of reproductive phasiRNAs—premeiotic 24-nt phasiRNAs—that are present in the *Zea* genus, including all five maize inbred lines and three teosinte species/subspecies that we examined, plus rice. We show that in the *Zea* genus, the premeiotic 24-nt phasiRNAs are distinct from the meiotic 24-nt phasiRNAs in triggering mechanism, effector protein, and molecular function.

Two size classes of reproductive, phased, small interfering RNAs (phasiRNAs)—21-nucleotides (nt) and 24-nt in length—accumulate to high abundance in the anthers of numerous flowering plants. The total abundance of the 21-nt phasiRNAs usually peaks at the premeiotic phase of anther development, whereas the 24-nt phasiRNAs peak during meiosis (1). The premeiotic 21-nt phasiRNAs and meiotic 24-nt phasiRNAs are derived from long noncoding loci and produced via two genetically separable biogenesis pathways. In both pathways, phasiRNA precursors (aka *PHAS* precursors) are generated from *PHAS* loci by RNA polymerase II, cleaved by an Argonaute1 (AGO1) clade protein directed by a microRNA (miRNA), converted to double-stranded RNA molecules, and chopped by a Dicer-like (DCL) protein to produce siRNAs that map to the corresponding genomic loci in a precise head-to-tail arrangement (hence the term “phased siRNA”). The two pathways differ primarily in miRNA triggers and DCL proteins, with miR2118 and DCL4 acting in the 21-nt phasiRNA pathway, and miR2275 and DCL5 acting in the meiotic 24-nt phasiRNA pathway (2). The AGO proteins known to load 21-nt phasiRNAs and 24-nt phasiRNAs include a few pathway-specific members and a few that are common to both pathways. For example, AGO1d loads 21-nt phasiRNAs in rice (3, 4); AGO5 clade proteins load 21-nt phasiRNAs in rice and maize (5, 6); and AGO18b loads both 21-nt and 24-nt phasiRNAs in maize (7).

Reproductive phasiRNAs are crucial for maintaining male fertility. Mutations in a few rice 21-nt phasiRNA loci (aka *21-PHAS* loci) cause temperature/photoperiod-sensitive male sterility (8, 9), and loss of a subset of *MIR2118* genes in rice causes male and female sterility (10). In maize, loss-of-function mutants of *Dcl5*, which likely functions specifically in the 24-nt phasiRNA pathway, exhibit temperature-sensitive male sterility (11). In terms of molecular functions, the 21-nt phasiRNAs are known to mediate *cis*-cleavage of their own precursors in rice and maize (12), and *trans*-cleavage of protein-coding mRNAs in rice (13, 14). In maize, 24-nt phasiRNAs are essential for maintaining CHH DNA methylation at their own genomic loci *in cis* (15). However, RNA targets of the 21-nt and 24-nt phasiRNAs in maize, if any, remain largely unknown.

The 21-nt phasiRNAs and meiotic 24-nt phasiRNAs both have been demonstrated to be widely present in angiosperms (16). However, their patterns of conservation differ; except for a handful of eudicot species that have recently been shown to accumulate 21-nt phasiRNAs (17), the majority of eudicots lack 21-nt phasiRNAs, whereas the meiotic 24-nt phasiRNAs are more broadly present in angiosperms. Notably, several eudicot species–including the model plant species *Arabidopsis thaliana*–apparently lack both 21- and 24-nt reproductive phasiRNAs (16). We previously reported a group of 24-nt phasiRNAs that peak at the premeiotic phase of anther development in barley and wheat and lack miR2275 target sites (18). However, whether the premeiotic 24-nt phasiRNAs are also present in other grass lineages remains poorly understood, as do their biogenesis mechanisms and functions.

Here, we carried out a comprehensive survey of reproductive phasiRNA pathways in five maize inbred lines and three teosinte species/subspecies. The maize inbred lines were chosen from among the founders of the maize nested association mapping population, including a stiff-stalk (B73), a non-stiff-stock (Oh43), a popcorn (HP301), a sweet corn (Il14H), and a tropical (NC358) variety, each representing a major clade of modern maize inbred lines (19, 20), and the teosinte varieties included two subspecies—*Zea mays* ssp. *parviglumis* (TIL11) and *Z. mays* ssp. *mexicana* (TIL25)—that are known as progenitors of modern maize (21), and *Zea luxurians* (RIL003). We show that in all eight *Zea* varieties, a substantial subset of *24-PHAS* loci are highly expressed at the premeiotic phase of anther development. These premeiotic 24-nt phasiRNAs are distinct from the meiotic 24-nt phasiRNAs and premeiotic 21-nt phasiRNAs in several aspects of biogenesis mechanisms and functions, and thus constitute a unique class of reproductive phasiRNAs.

## Results

### Conservation of PhasiRNA Pathway Genes in *Zea* Genus.

**Fig. 1.**
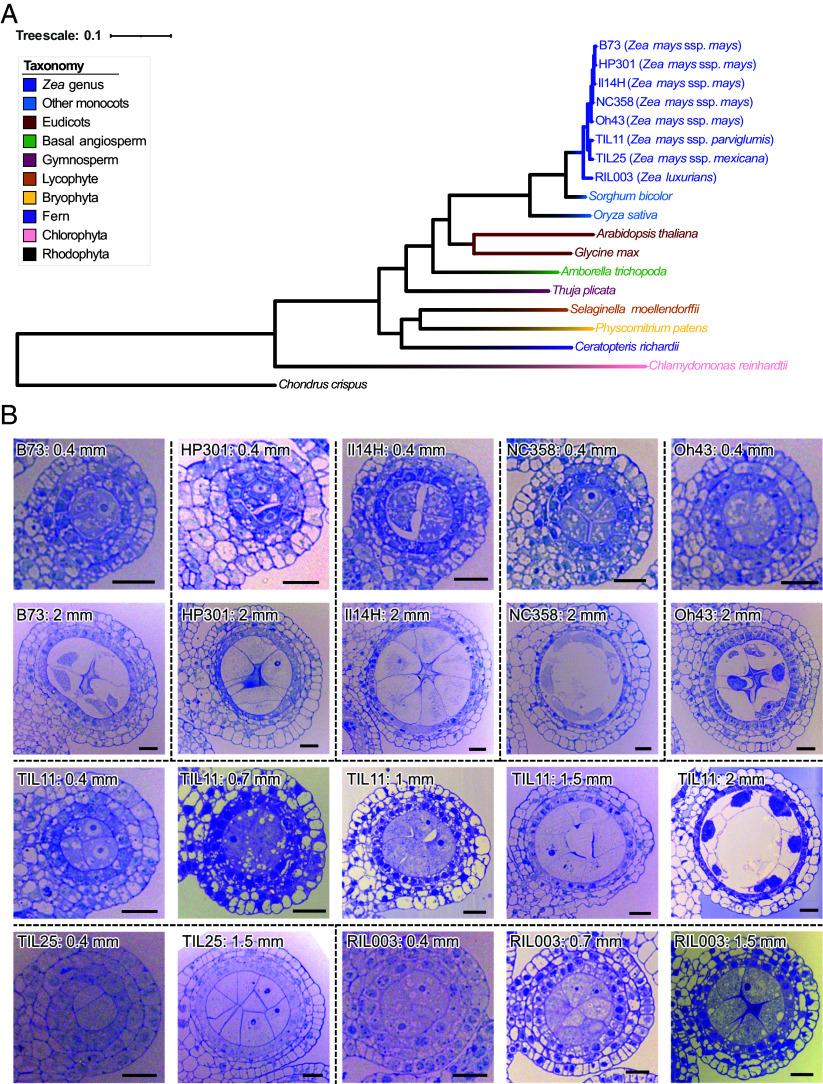
*Zea* varieties and anther developmental stages sampled for the comparative analysis of phasiRNAs. (*A*) Phylogeny of the *Zea* varieties and outgroup species. (*B*) Micrographs of cross sections of anthers representing those sampled for transcriptome and degradome analyses. In the maize varieties (B73, HP301, Il14H, NC358, and Oh43) and TIL25, 0.4-mm anthers are premeiotic and 2-mm anthers are meiotic. In TIL11, 0.4 to 0.7-mm anthers are premeiotic, and 1.5- to 2-mm anthers are meiotic. In RIL003, 0.4- and 0.7-mm anthers are premeiotic, and 1.5-mm anthers are meiotic. (Scale bars, 20 μm.)

To identify reproductive *PHAS* loci and phasiRNA pathway genes in the eight *Zea* varieties (Fig. 1*A*), we performed small RNA-seq (sRNA-seq), RNA-seq, and nanoPARE analyses of 2 to 5 developmental stages of anthers in each variety, spanning premeiotic to meiotic phases (Fig. 1*B*). We annotated the HD-ZIP IV, bHLH, RDR, DCL, AGO, SGS3, DRB, SE, SDN1, and HESO1/URT1 family proteins, which are involved in the biogenesis of phasiRNAs and/or miRNAs (2), encoded in all the *Zea* genomes and a few outgroup species, and performed phylogenetic analyses for each family (Fig. 1*A* and *SI Appendix*, Fig. S1). The gene copy numbers of each major clade in each phylogenetic tree are similar across the *Zea* genomes, with only a few clades (e.g. the RDR6, DRB4, and HEN1 clades) exhibiting copy number variation (Dataset S1). These data suggest that the phasiRNA pathway genes are largely conserved in the *Zea* genus.

### Identification of Reproductive *PHAS* Loci in the *Zea* Varieties.

**Fig. 2.**
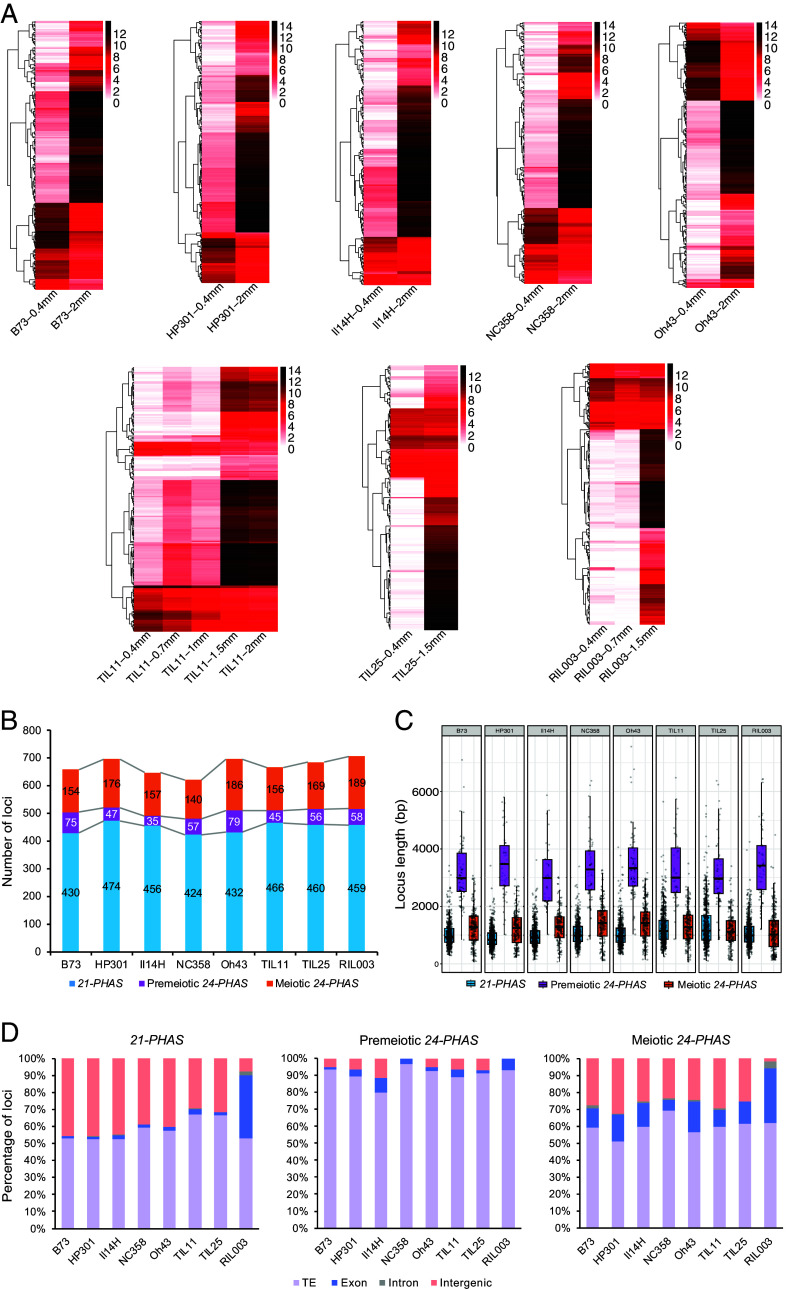
Identification of reproductive *PHAS* loci in the *Zea* varieties. (*A*) Heatmaps of abundance of phasiRNAs derived from individual *24-PHAS* loci. Heatmaps were clustered on Euclidean distance. (*B*) Numbers of *PHAS* loci in each genome. (*C*) Boxplot of *PHAS* loci lengths. (*D*) Percentages of *PHAS* loci overlapping with TEs, exons, introns, or intergenic regions.

Using the sRNA-seq data, we identified reproductive *PHAS* loci and examined their temporal expression patterns during early anther development in the *Zea* varieties. Similar to the W23 *bz2* maize (1), we found that in the five maize inbred lines plus TIL11, 21-nt phasiRNAs were more abundant in premeiotic anthers than in meiotic anthers. However, in TIL25 and RIL003, 21-nt phasiRNAs were more abundant at the meiotic phase than the premeiotic phase (*SI Appendix*, Fig. S2). These results suggest that the gene regulatory program controlling the temporal accumulation of 21-nt phasiRNAs diverged between TIL11 and the other two teosinte varieties, and the TIL11-like program was retained in modern maize. Remarkably, while it had previously been demonstrated in maize that the 24-nt phasiRNAs are most abundant during meiosis (1), in all the *Zea* varieties we analyzed, we observed a group of *24-PHAS* loci that already accumulated a high abundance of phasiRNAs at the premeiotic phase (Fig. 2*A* and Dataset S2). Hereafter, the *PHAS* loci that produce substantial levels of phasiRNAs [counts per million (CPM) > 20] at the premeiotic stage are referred to as premeiotic *24-PHAS* loci, and the other *24-PHAS* loci are referred to as meiotic *24-PHAS* loci. The two classes of *24-PHAS* loci and the *21-PHAS* loci do not overlap in genomic locations in any of the *Zea* varieties. In TIL11 and the modern maize varieties, phasiRNA abundance of the premeiotic *24-PHAS* loci declined substantially by the meiotic phase, whereas this decrease did not occur or was modest in TIL25 and RIL003 (Fig. 2*A*), suggesting that TIL11 evolved a regulatory mechanism that down-regulates the abundance of 24-nt phasiRNAs after the premeiotic phase, and such a mechanism has been maintained in modern maize.

The numbers of *21-* and *24-PHAS* loci were similar across the *Zea* varieties and to the previously reported loci numbers in W23 *bz2*, which has 463 *21-PHAS* and 176 *24-PHAS* loci (1) (Fig. 2*B*). Interestingly, in every variety, the premeiotic *24-PHAS* loci were significantly longer than the *21-PHAS* and meiotic *24-PHAS* loci (*P* < 0.05) (Fig. 2*C* and Dataset S3). The distribution of *PHAS* loci across chromosomes was similar among the *Zea* varieties in that i) in every variety, the *21-PHAS*, premeiotic *24-PHAS*, and meiotic *24-PHAS* were distributed across all chromosomes (*SI Appendix*, Figs. S3 and S4); and ii) in nearly every variety, Chr. 2 had the largest number of *21-PHAS* loci, Chr. 4 had the largest number of premeiotic *24-PHAS* loci, and Chr. 1 had the largest number of meiotic *24-PHAS* loci (*SI Appendix*, Fig. S3).

For both premeiotic and meiotic *24-PHAS* loci, the major sRNAs were enriched for a 5′-terminal adenine (5′-A). For the *21-PHAS* loci, in all eight varieties, the 19th nucleotide position of the major sRNAs was consistently enriched for cytosine (C) and underrepresented by A, while the 20th position was enriched for uracil (U) and underrepresented by C. On the other hand, the major sRNAs of the *21-PHAS* loci were enriched for a 5′-A in B73, HP301, NC358, and Oh43, but were enriched for a 5′-C in Il14H, TIL11, TIL25, and RIL003 (*SI Appendix*, Fig. S5). Prior work in rice demonstrated that the rice AGO5c [aka MEIOSIS ARRESTED AT LEPTOTENE1 (MEL1)] preferentially loads sRNAs with a 5′-C (5). Whether the differential enrichment of the 5′ end nucleotide of 21-nt phasiRNAs in the *Zea* varieties affects their sorting onto AGO proteins is yet to be determined.

In all *Zea* varieties, the majority of *PHAS* loci overlapped with transposons (Fig. 2*D* and Dataset S4). The majority of transposons that overlapped with *21-PHAS* loci were terminal inverted repeats (TIRs), which are DNA transposons, whereas the majority of those that overlapped with *24-PHAS* loci—both premeiotic and meiotic loci— were long terminal repeats (LTRs), which are retrotransposons (*SI Appendix*, Fig. S6 *A–C*). Furthermore, *21-PHAS* overlapped primarily with the PIF/Harbinger-type TIRs, whereas *24-PHAS* overlapped primarily with the Gypsy-type LTRs (*SI Appendix*, Fig. S6 *D*–*F*). These results indicate distinct genomic origins of the 21- and 24-nt phasiRNAs, and suggest that the two size classes of reproductive phasiRNAs may play different roles in transposon silencing (if that is their biological function). Notably, in RIL003, 37% of the *21-PHAS* loci and 32.3% of the meiotic *24-PHAS* loci overlapped with exons (Fig. 2*D* and Dataset S4). These proportions are much higher than in the other varieties and is possibly due to the fact that the RIL003 transcriptome was assembled and annotated using our anther data and with different methods than those used for annotating the other *Zea* genomes.

**Fig. 3.**
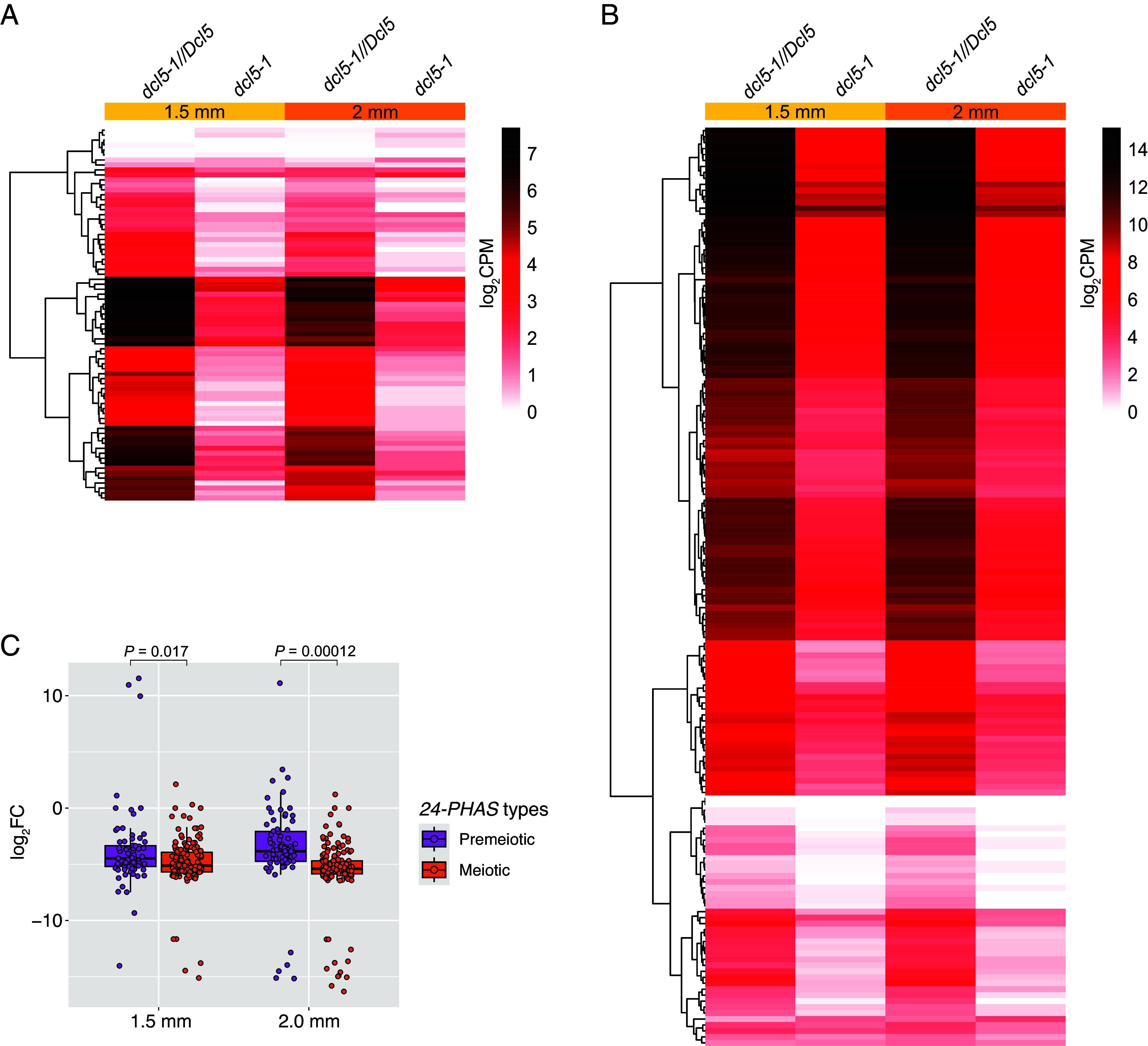
Abundance of premeiotic and meiotic 24-nt phasiRNAs in the previously reported *dcl5-1* mutant compared to heterozygous siblings (*dcl5-1//Dcl5*) (11). (*A* and *B*) Abundance of 24-nt sRNAs derived from premeiotic (*A*) and meiotic (*B*) *24-PHAS* loci. In both heatmaps, each row represents a *24-PHAS* locus. Heatmaps were clustered on Euclidean distance. (*C*) Box plot of log_2_FC of the abundance of 24-nt phasiRNAs derived from premeiotic and meiotic *24-PHAS* loci. The *P* values were calculated using unpaired Student’s *t* test.

Interrogation of a previously published sRNA-seq dataset of a null *dcl5* mutant (11) demonstrated that the majority of both the premeiotic and meiotic *24-PHAS* loci were down-regulated substantially in *dcl5* (Fig. 3), suggesting that both types of 24-nt phasiRNAs are dependent on DCL5. In support of this notion, *DCL5* is expressed in the premeiotic anthers of wild-type maize, although at lower levels compared to the 1.5-mm stage (*SI Appendix*, Fig. S7) (22). Notably, many *24-PHAS* loci of both types still produce detectable levels of 24-nt phasiRNAs in *dcl5* (Fig. 3 *A* and *B*), indicating that *24-PHAS* precursors are processed by another DCL protein in the absence of DCL5. This DCL protein is possibly DCL3, a paralog of DCL5 and known to process 24-nt siRNAs (2). Moreover, the downregulation of meiotic 24-nt phasiRNAs was significantly more substantial than the premeiotic 24-nt phasiRNAs at both 1.5- and 2-mm stages (*P* < 0.05; Fig. 3*C*), suggesting that the meiotic 24-nt phasiRNAs are more crucially dependent on DCL5 than the premeiotic 24-nt phasiRNAs. Using RNA-seq data from the prior DCL5 study (11), we performed a differential transposon expression analysis of 1.5-mm anthers from the *dcl5* mutant but did not detect any differentially expressed transposons [fold change (FC) > 1.5, FDR < 0.05; Dataset S5], suggesting that both classes of 24-nt phasiRNAs are unlikely to transcriptional repress transposons.

Premeiotic 24-nt phasiRNAs have been reported in barley and wheat, but not in maize or rice (18). Thus, we identified *24-PHAS* loci in rice and examined their temporal expression patterns using two previously published sRNA-seq datasets (3, 23) using the same methods we applied to our *Zea* data. We found that 34 of the 79 *24-PHAS* loci in rice already produced abundant phasiRNAs at premeiotic stages (*SI Appendix*, Fig. S8 and Dataset S6). Similar to the *Zea* varieties, the rice premeiotic *24-PHAS* loci are significantly longer than rice *21-PHAS* and meiotic *24-PHAS* loci (*SI Appendix*, Fig. S9). Therefore, we conclude that premeiotic 24-nt phasiRNAs are more broadly present in grasses than previously thought.

### Identification of miRNA Loci in the *Zea* Genomes.

Using ShortStack (24), miR-PREFeR (25), and miRador (26), we annotated the expressed miRNAs in the *Zea* genomes. A union of miRNAs identified by the three methods yielded 175 (in NC358) to 366 (in TIL11) mature miRNA sequences encoded by each genome (*SI Appendix*, Fig. S10 and Dataset S7). In every variety, we detected expression of at least one copy of *MIR2118* and *MIR2275* in the anthers (Dataset S8), consistent with their canonical roles in triggering reproductive phasiRNAs. To identify all putative *MIR2118* and *MIR2275* loci in the *Zea* genomes, including those without detectable mature miRNAs in our anther samples, we employed a BLAST-based approach to identify all putative *MIR2118*/*MIR2275* loci in the eight genomes, without relying on sRNA-seq reads. This led to identification of 15 to 17 *MIR2118* loci and 6 to 11 *MIR2275* loci in each genome (Dataset S9). The numbers of *MIR2118/MIR2275* copies and their chromosomal distributions are similar across the *Zea* varieties suggesting high levels of functional conservation of the two miRNA families in the *Zea* genus.

### The Premeiotic 24-nt PhasiRNAs Are Unique in Triggering Mechanism and Targets.

To identify the triggers of reproductive phasiRNAs in the *Zea* varieties, we predicted interactions between miR2118 and *21-PHAS* precursors and between miR2275 and *24-PHAS* precursors using sPARTA (27). In each *Zea* variety, 64.1 to 84.7% of the *21-PHAS* precursors have a predicted miR2118 target site, and 66.1 to 79.0% of the meiotic *24-PHAS* have a predicted miR2275 target site, respectively. In contrast, only 3.4 to 17.0% of the premeiotic *24-PHAS* have a predicted miR2275 target site (Dataset S10). Consistent with these predicted miRNA-*PHAS* interactions, an enrichment analysis of ~22-nt motifs in the *PHAS* loci of each *Zea* variety detected a miR2118-matching motif in the *21-PHAS* loci and a miR2275-matching motif enriched in the meiotic *24-PHAS* loci, whereas no miR2275-like motif was found to be enriched in the premeiotic *24-PHAS* loci (Dataset S11). Moreover, none of the 20- to 22-nt motifs enriched in the premeiotic *24-PHAS* loci were similar to known maize miRNAs in sequences (Dataset S11), suggesting that the maize premeiotic 24-nt phasiRNAs are not triggered by miRNAs.

To validate the phasiRNA triggers in the *Zea* varieties, we performed nanoPARE (28) analyses on the same anther materials we used for sRNA-seq. In line with our predictions above, many *21-PHAS* precursors and meiotic *24-PHAS* precursors were targeted by miR2118 or miR2275 (Dataset S12, Sheets 1 and 3 to 22). In TIL11, we observed the largest number of miR2118–*21-PHAS* interactions at 0.7 mm compared to the other stages, and the largest number of miR2275–*24-PHAS* at 1.5 mm (Dataset S12, Sheet 1), indicating stage-specific biogenesis of reproductive phasiRNAs. Notably, premeiotic *24-PHAS* precursors were rarely targeted by miR2275 or any other known miRNAs based on our nanoPARE analysis (Dataset S12, Sheet 1). This result confirms that the premeiotic 24-nt phasiRNAs are likely not triggered by miRNAs.

Using the nanoPARE data, we also detected several miRNA-mediated mRNA cleavage events in each *Zea* variety (Dataset S12, Sheet 2). Notably, several of the miRNA families mediate cleavage of mRNAs encoded by orthologous genes in several of the *Zea* genomes. For example, miR160, miR171, and miR396 each regulates one or two genes that are highly conserved (i.e., within a pangene set) across all *Zea* varieties with available pangene annotation (i.e., except RIL003), plus several other genes that are in a pangene set comprising fewer *Zea* genomes. The highly conserved miRNA-target pairs are likely to play a key role in regulating anther development.

An analysis of phasiRNA targets showed that, in every *Zea* variety, phasiRNAs regulate a small number of protein-coding transcripts, and only a few targets of the meiotic 24-nt phasiRNAs, belonging to two pangene sets, are conserved in three or four *Zea* varieties (Dataset S13, Sheet 2). These results suggest that either the majority of reproductive phasiRNAs function via a mechanism other than mediating mRNA cleavage or they act on only a small number of target genes that diverged rapidly in the *Zea* genus. Consistent with a prior study in maize and rice (12), 21-nt phasiRNAs can mediate *cis*-cleavage in all *Zea* varieties (Dataset S13, Sheets 1 and 3 to 22). Our analyses also detected many *21-PHAS* precursors that are targeted by 21-nt phasiRNAs *in trans* (Dataset S13, Sheet 1). Furthermore, meiotic 24-nt phasiRNAs also mediate *cis*-cleavage and/or *trans*-cleavage of other *24-PHAS* precursors in most of the varieties (Dataset S13, Sheet 1). In contrast, the premeiotic 24-nt phasiRNAs do not mediate *cis*- or *trans*-cleavage of *PHAS* precursors in nearly all the varieties with the exception of two *cis*-cleavage events detected in B73). These results suggest that the premeiotic 24-nt phasiRNAs are distinct from the meiotic 24-nt phasiRNAs (and the 21-nt phasiRNAs) in their abilities to mediate *PHAS* precursor cleavage.

### *AGO18* Genes Are Essential for Normal Accumulation of Meiotic 24-nt PhasiRNAs But Not the Premeiotic 24-nt PhasiRNAs.

AGO18b is known to load 21- and 24-nt phasiRNAs in maize (7), and in rice, the single-copy *AGO18* gene has been shown to be crucial for male fertility (29). To understand the role of the *AGO18* genes in maize anther development and phasiRNA pathways, we generated a triple knockout mutant of all three *AGO18* copies (*SI Appendix*, Fig. S1*E*) by crossing an *ago18a;b* double null mutant that we generated previously using CRISPR-Cas9 (30) with a *ago18c* null mutant obtained from the Mu-Illumina population (31) (*SI Appendix*, Fig. S11). The triple homozygous mutant exhibited no obvious defect in fertility, forming fertile tassels and anthers, viable pollen grains, and fully pollinated ears when self-pollinated (*SI Appendix*, Fig. S12), suggesting that the *AGO18* genes are dispensable for male and female fertility under normal growth conditions.

**Fig. 4.**
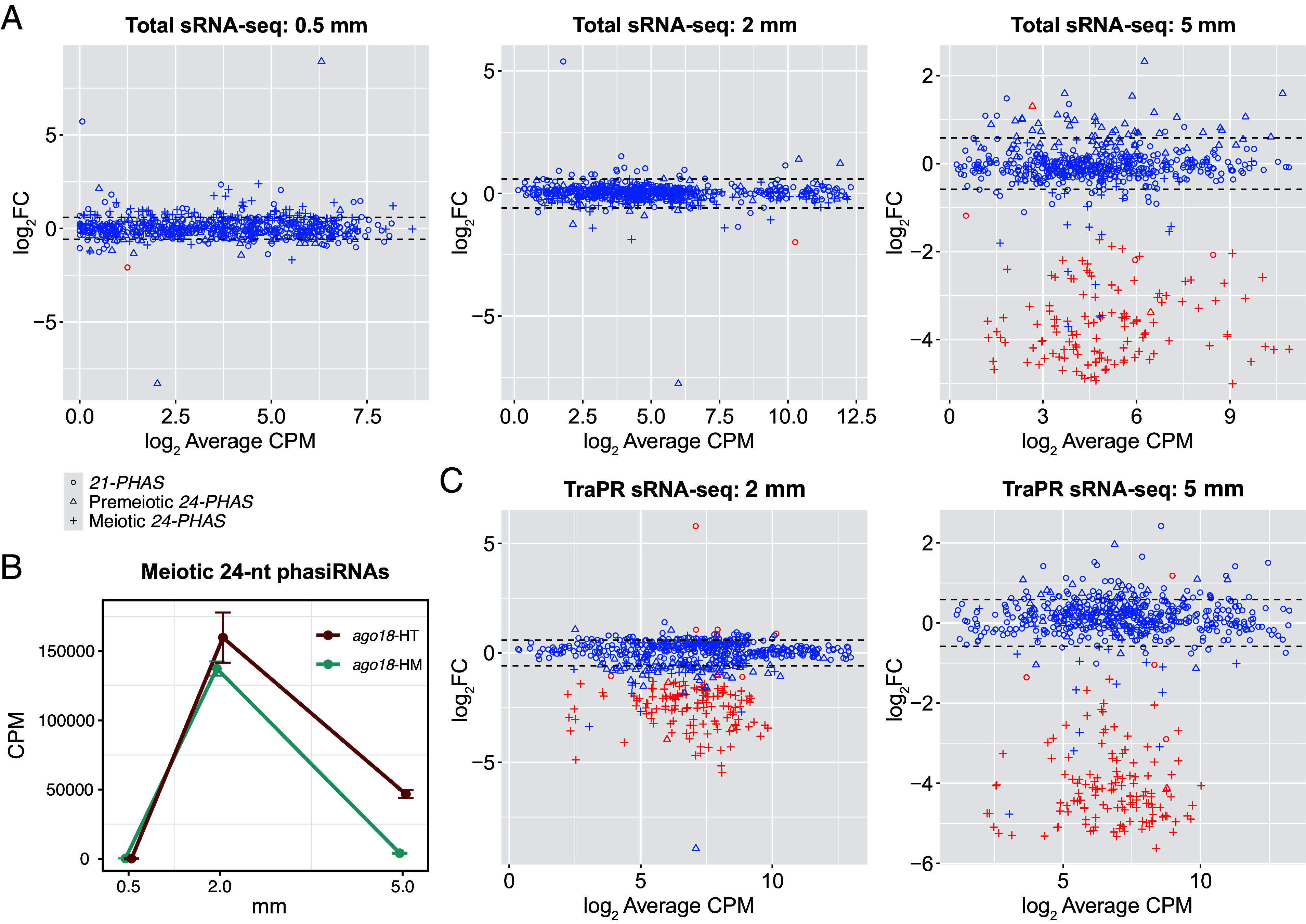
Total sRNA-seq and TraPR sRNA-seq analyses of the *ago18* triple mutant anthers. (*A*) Mean-difference (MA) plots of *PHAS*loci based on sRNA-seq. (*B*) Total abundance (mean ± SE) of meiotic 24-nt phasiRNAs in *ago18* triple homozygous mutant plants (*ago18*-HM) and their triple heterozygous siblings (*ago18*-HT). Results of Student’s *t* test are shown in *SI Appendix*, Fig. S13. (*C*) MA plots from differential accumulation analyses of phasiRNA abundance per loci based on TraPR sRNA-seq. In *A* and *C*, average CPM values were calculated for only triple heterozygous samples. Significantly differentially expressed loci (FC > 1.5, FDR < 0.05) are represented by red circles/triangles/plus signs, and the other loci in blue. Dash lines indicate FC = ±1.5 (i.e., log_2_FC = ±0.585).

Our RNA-seq analyses of developing anthers detected only 47 genes differentially expressed between the triple homozygous mutant plants and their triple heterozygous siblings at 0.5 mm (premeiotic), one gene differentially expressed at 2 mm (meiotic), and none at 5 mm (postmeiotic) (FC > 1.5, FDR < 0.05; Dataset S14), suggesting that loss of *AGO18* genes has a minor impact on the anther mRNA transcriptome. Using sRNA-seq, we detected, at the 0.5- and 2-mm stages, only one *21-PHAS* locus that was differentially expressed between the *ago18* triple mutant and its triple heterozygous siblings, whereas at the 5-mm stage, three *21-PHAS*, two premeiotic *24-PHAS*, and 123 meiotic *24-PHAS* loci were significantly down-regulated in the mutant (Fig. 4*A* and Dataset S15). Accordingly, by the 5-mm stage, the total abundance of meiotic 24-nt phasiRNAs was also down-regulated dramatically, with average CPM decreasing from 46,664.8 to 3,987.3 (FC = 11.7; *P* = 3.8 × 10^−3^) (Fig. 4*B*). In contrast, the total abundance of the 21-nt phasiRNAs or premeiotic 24-nt phasiRNAs was not down- or up-regulated significantly at any of the three stages (*SI Appendix*, Fig. S13). In addition, we detected only three differentially accumulated miRNAs, including a putative miR159 and a putative miR2275 (Dataset S16). Together, these sRNA-seq results suggest that the *AGO18* genes play a key role in the normal accumulation of meiotic 24-nt phasiRNAs and a minor role in that of the 21-nt phasiRNAs, premeiotic 24-nt phasiRNAs, and miRNAs.

To characterize the impact of loss of *AGO18* genes on the profile of AGO-loaded sRNAs, we carried out high-throughput sequencing of sRNAs isolated using the TraPR method (32) from 2- and 5-mm anthers of the *ago18* triple mutant. In contrast to the total sRNA-seq results described above, 98 meiotic *24-PHAS* loci, two premeiotic *24-PHAS* loci, and two *21-PHAS* loci were already down-regulated in phasiRNA abundance at 2 mm. At the 5-mm stage, 129 meiotic *24-PHAS* loci, one premeiotic *24-PHAS*, and four *21-PHAS* loci were down-regulated in the mutant (Fig. 4*C* and Dataset S17). Moreover, the total abundance of meiotic 24-nt phasiRNAs demonstrated significant downregulation at 2 and 5 mm, whereas the premeiotic 24-nt phasiRNAs did not show this (*SI Appendix*, Fig. S14). Together, our total sRNA-seq and TraPR sRNA-seq analyses indicate that, at 2 mm, the loss of *AGO18* genes does not affect the abundance of meiotic 24-nt phasiRNAs, but significantly fewer meiotic 24-nt phasiRNAs are loaded onto AGOs, suggesting that AGO18 proteins are essential for loading meiotic 24-nt phasiRNAs but not for their accumulation at the 2-mm stage. In contrast, at the 5-mm stage, AGO18 proteins are essential for both loading and accumulation of 24-nt phasiRNAs.

NanoPARE analyses of 0.5-, 2-, and 5-mm anthers of the *ago18* triple mutant demonstrated that for both miRNAs and reproductive phasiRNAs, the sRNA-target interactions were consistently fewer in the mutant compared to their heterozygous siblings (Dataset S18), suggesting that AGO18 facilitates miRNA and phasiRNA-mediated gene silencing. Furthermore, at 0.5 and 2 mm, we detected many *trans*-cleavage events of *PHAS* precursors by 21-nt and meiotic 24-nt phasiRNAs, consistent with our analysis of wild-type maize varieties, yet most of the cleavage events were not detected in the triple mutant (Dataset S18), suggesting that the loading of 21-nt phasiRNAs and meiotic 24-nt phasiRNAs by AGO18 is crucial for *cis*- and *trans*-cleavage of reproductive *PHAS* precursors.

## Discussion

We demonstrate that premeiotic 24-nt phasiRNAs are present in all the *Zea* varieties that we analyzed—including five maize inbred lines and three teosinte species/subspecies —and rice (Fig. 1 and *SI Appendix*, Fig. S8). In the *Zea* genus, premeiotic 24-nt phasiRNAs exhibited several similarities and differences compared to meiotic 24-nt phasiRNAs. The two types of 24-nt phasiRNAs are similar in that i) their genomic origins, which overlap predominantly with transposons (Fig. 2*D* and *SI Appendix*, Fig. S6); and ii) their dependence on DCL5 for biogenesis (Fig. 3). However, the premeiotic 24-nt phasiRNAs are unique in several aspects. First, their genomic loci are distinct and significantly longer than those of the 21-nt and meiotic 24-nt phasiRNAs (Fig. 2*C*). Second, they are likely not triggered by miRNA-mediated cleavage of *PHAS* precursors, but rather by an unknown mechanism (Dataset S11). Third, the vast majority of premeiotic 24-nt phasiRNAs are likely not loaded by AGO18, whereas many meiotic 24-nt phasiRNAs are (Fig. 4). Fourth, while both the 21-nt phasiRNAs and the meiotic 24-nt phasiRNAs can mediate cleavage of *PHAS* precursors, the premeiotic 24-nt phasiRNAs generally do not mediate *PHAS* precursor cleavage (Dataset S13). Together, these findings indicate that the premeiotic 24-nt phasiRNAs constitute a unique class of reproductive phasiRNAs with respect to both biogenesis mechanism and molecular function. We speculate that a possible reason that the premeiotic 24-nt phasiRNAs were not identified in previous work (1) was that the anther sRNA data were generated from the W23 *bz2* inbred line, but were aligned to the B73 reference genome for analyses, requiring zero mismatch in identifying *PHAS* loci. Thus, mapping sRNA data to the corresponding genome might be crucial; this is easier today than it was in 2015 because of the proliferation of high-quality genomes.

Our observations that only a small number of anther-expressed protein-coding genes are down-regulated in the *ago18* triple mutant (Dataset S14) but many *24-PHAS* loci are down-regulated in sRNA abundance (Fig. 4*A* and Dataset S15) suggest that in anthers the *AGO18* genes function predominantly in the reproductive phasiRNA pathways. The substantial differences we observed in total sRNA-seq versus TraPR sRNA-seq analyses of the *ago18* mutant—especially at the 2-mm stage (Fig. 4 *A* and *C*)—indicate that the meiotic 24-nt phasiRNAs can accumulate in maize anthers without being loaded onto an AGO protein, at least for a period of time. However, by the postmeiotic phase of anther development, the AGO18 proteins seem necessary for normal phasiRNA accumulation (Fig. 4*A*). This in turn suggests that the meiotic 24-nt phasiRNAs may have crucial functions during postmeiotic anther development. Nonetheless, the lack of an obvious phenotype of the *ago18* triple mutant (*SI Appendix*, Fig. S12), contradicting a prior report showing that the rice *AGO18* gene is crucial for male fertility (29), suggests that perhaps in maize other AGO protein(s) act redundantly or in a compensatory manner with AGO18 in the phasiRNA pathways.

In summary, this work demonstrates that the premeiotic 24-nt phasiRNAs are more broadly present—at least in the grass lineage—than previously thought. Our data provide significant insights into the biogenesis and molecular function of the premeiotic 24-nt phasiRNAs, including the roles of several biogenesis factors or effector proteins such as DCL5 and AGO18. However, the identity of the other key players in the premeiotic 24-nt phasiRNA pathway, and whether premeiotic 24-nt phasiRNAs exist in other grass species and more broadly across monocots, remains unknown. Therefore, more work will be needed to further understand the biogenesis, function, and evolution of these premeiotic 24-nt phasiRNAs.

## Materials and Methods

### Plant Materials and Growth.

Seeds of HP301, Il14H, NC358, Oh43, and TIL11 were provided by Matthew Hufford (Iowa State University), TIL25 seeds were provided by John Doebley (University of Wisconsin-Madison), and RIL003 seeds were obtained through the US National Plant Germplasm System (accession: PI 422162). The *ago18* triple mutant was generated by crossing a previously reported *ago18a;b* double mutant (30) with an *ago18c* mutant obtained from the Mu-Illumina population (insertion ID: mu-illumina_50570.7) (31); the *ago18* materials analyzed in this work are transgene-free. Maize plants were grown in greenhouses of the Donald Danforth Plant Science Center Plant Growth Facility under 14-h day length, 28 °C/24 °C temperature cycles, and 50% humidity, and the teosinte varieties were grown in a walk-in growth chamber at the same facility under 12-h day length, 28 °C/23 °C temperature cycles, and 40% humidity.

### Cytological Analysis.

Fresh anthers were fixed with 2% [v/v] paraformaldehyde, 2% [v/v] glutaraldehyde, and 0.1% [v/v] Tween-20 in 0.1 M PIPES [piperazine-*N,N'*-bis(2-ethanesulfonic acid)] buffer (pH 7.4) overnight, dehydrated using a concentration gradient of acetone (30%, 50%, 70%, 80%, 90%, and 100% [v/v]), embedded using a Quetol 651 - NSA Kit (no. 14640, Electron Microscopy Sciences), and polymerized at 60 °C. Embedded tissues were sectioned into 500 nm sections using a Leica Ultracut UCT (Leica Microsystems Inc.), and stained using the Epoxy Tissue Stain solution (no. 14950, Electron Microscopy Sciences). Anther sections were imaged using a Leica DM 750 microscope. Images were captured with a Leica ICC50 HD camera and Leica Acquire v2.0 software (Leica Microsystems Inc.).

### Library Preparation and Sequencing.

Fresh anther samples, each with 2 or 3 biological replicates derived from distinct plants, were snap-frozen in liquid nitrogen, and total RNA was extracted using the TRI reagent (Sigma-Aldrich) or the TraPR Small RNA Isolation kit (Lexogen; for TraPR sRNA-seq only). sRNA-seq libraries were prepared from ~100 ng total RNA per sample using a Somagenics RealSeq-AC miRNA library kit following the manufacturer’s protocol. RNA-seq (Smart-seq2) and nanoPARE libraries were prepared from 5 ng total RNA per sample using the nanoPARE library preparation protocol (28) with previously described modifications (17). All libraries were sequenced on an Illumina NextSeq 550 instrument at the University of Delaware DNA Sequencing & Genotyping Center to generate 76-nt single-end reads.

### Whole Proteome-Based Phylogenetic Analysis.

The anther transcriptome of RIL003 was assembled from the RNA-seq and nanoPARE reads. Briefly, StringTie v2.1.7 (33) and Scallop v0.10.4 (34) were separately used with default parameters to perform de novo transcriptome assembly, and the resulting transcriptome annotations were merged using the merge function of StringTie. Protein-coding transcripts were identified using TranSuite v0.2.2 (35) with parameter “Auto”. RIL003 transposons were annotated using Extensive de novo TE Annotator v2.0.1 (36) with parameters --species Maize --anno 1 --force 1. Annotation of gene models and transposons of all the other *Zea* genomes (B73 RefGen_v5 and v1 of all the other varieties) were obtained from maizeGDB (37). Whole proteome sequences of the outgroup species were obtained from Ensembl Plants (*Chondrus crispus*) or Phytozome v13 (all the other species). A species tree was generated using Orthofinder v2.5.4 (38) with default parameters. Gene trees were built using previously described methods (39).

### *PHAS* Loci Identification and Analyses.

*PHAS* loci were identified from sRNA-seq data using ShortStack v3.8.5 with parameters --mismatches 0 --mincov 0.5 rpm and phasing scores ≥ 30 as the cutoff. The length of each *PHAS* precursor, as estimated by ShortStack, was used as a proxy to the length of the *PHAS* locus. Premeiotic *24-PHAS* loci were defined as those with a minimal CPM (mean of replicates) >20 at premeiotic stages, and the remaining *24-PHAS* loci were defined as meiotic. Rice sRNA-seq were obtained from two previous studies (3, 23). Data from ref. 3 were used to identify *PHAS* loci, and data from both studies were separately normalized and analyzed for abundance of 24-nt phasiRNAs per loci. Premeiotic *24-PHAS* loci were defined as those with minimal CPM > 20 at premeiotic stages based on data from ref. 23. Overlap among the three types of *PHAS* loci or between *PHAS* loci and various genomic features were determined using the intersect function of BEDtools v2.29.2 (40) with parameters -e -f 0.5 -F 0.5.

### miRNA Loci Identification and Analyses.

To annotate expressed miRNA loci, all sRNA-seq data for each genotype were analyzed using ShortStack v3.8.5 with parameters --mismatches 0 --dicermax 22 --mincov 15, miR-PREFeR v0.24 with parameters PRECURSOR_LEN = 300, READS_DEPTH_CUTOFF = 20, MIN_MATURE_LEN = 20, MAX_MATURE_LEN = 22, ALLOW_NO_STAR_EXPRESSION = N, ALLOW_3NT_OVERHANG = N, CHECKPOINT_SIZE = 300, and miRador with default parameters. Using BLASTN, miRNAs were assigned to known miRNA families in miRBase (41) release 22.1 (≤5 mismatches) or defined as previously unannotated miRNAs (requiring corresponding miRNA* reads). For each genome, a union of all the expressed mature miRNAs identified using the three tools was used for downstream analyses.

To identify all putative *MIR2118* and *MIR2275* loci in the *Zea* genomes, publicly available *MIR2118*/*MIR2275* precursor sequences were obtained from miRBase and a previous study (42) and used as queries to search for homologous sequences in the *Zea* genomes using BLASTN with parameters -max_target_seqs 10 -evalue 10 -word_size 10. The subject regions that were longer than 80% of the length of query sequences were filtered and merged using the merge function of BEDtools. The resulting sequences were aligned using MUSCLE (43), and phylogenetic trees were built using IQ-TREE v2.2.0.3 (44) to assign/curate names of *MIR2118* and *MIR2275* loci based on orthology. MUSCLE-generated alignments were examined using Jalview v2 (45) to identify putative mature miR2118/miR2275 sequences and corresponding miRNA* sequences that are homologous to known members of the families.

### sRNA/mRNA Quantification and Differential Expression Analyses.

To quantify phasiRNA abundance, sRNA-seq reads were mapped to the respective *Zea* genomes using Bowtie v1.3.1 (46); to quantify miRNA abundance, sRNA-seq reads were mapped to a FASTA file of all mature miRNAs annotated for each genome using Bowtie; and to quantify mRNA abundance, RNA-seq/Smart-seq2 reads were mapped to the respective *Zea* genomes (B73 RefGen_v5 was used for the *dcl5* and *ago18* mutant data) using HISAT v2.1.0 (47) with parameters --min-intronlen 30 --max-intronlen 8000. Reads mapped to *PHAS* loci or miRNAs were counted using featureCounts v1.6.3 (48) with parameter -M and normalized to CPM using edgeR v4.0.2 (49). Differential expression analyses were performed using edgeR with a generalized linear model–based method.

### Small RNA Target Identification.

Small RNA-transcript interactions in each anther sample were identified using the nanoPARE analysis pipeline (28). The sRNA-target pairs with adjusted *P* value < 0.05 at the EndCut step and are detected in at least two biological replicates were considered positive.

### Identification of RNA Polymerase II-Derived Strands of *PHAS* Precursors.

NanoPARE reads mapped to both strands of genomic DNA were counted separately using featureCounts for each *PHAS* locus. For loci with >10 raw reads, the RNA strand that accounts for >70% of the total reads was considered as the RNA polymerase II-derived strand of a precursor.

### Motif Enrichment analysis.

For detection of potential miRNA-target sites in *PHAS* precursors, *PHAS* loci were extended by 500 bp on both ends. The sequences of extended *PHAS* loci were obtained using the getfasta function of BEDTools, and motif enrichments were detected using the Multiple Expectation maximizations for Motif Elicitation (MEME) program (*E* value < 0.05) of the MEME Suite (50). Enriched motifs were aligned with known maize miRNA sequences from miRBase using the Tomtom program (*E* value < 0.001) of the MEME Suite.

### Quantification of Male Fertility.

Anther exertion was monitored daily, and tassels were detached on the day when anthers in the lower florets of the lowest tassel branch exerted. Red/green/blue (RGB) images of the tassels were captured using a digital single-lens reflex camera controlled by a Raspberry Pi within an enclosed light tent to ensure consistent imaging settings. Four photos were taken for each tassel with 90° rotations to capture variations from different angles. The Tasselyzer pipeline (51) was applied to the tassel images to quantify anther exertion. Images were analyzed at the pixel level based on color information and segmented into anthers, other tassel areas, and background, using a naïve Bayes classifier on the PlantCV platform (52). The ratio of anther pixels to the total pixels of anther and other tassel areas was calculated to quantify male fertility mimicking visual observations. Anther/tassel area ratios were averaged among the four images taken from different angles. Fuchsia and green highlights were applied to anthers and other tassel areas for visual inspection of segmentation results and demonstration purposes.

### Pollen Imaging.

Anthers were dissected shortly before exertion and placed in a 1.7 mL tube. About 6 anthers were stained in 35 µL of Alexander staining solution for 5 min. During staining, anthers were gently squeezed using forceps to release pollen grains. The tube was centrifuged at 100 × g for 1 min, and the debris of somatic cells was removed using forceps. Pollen grains were washed twice in 100 µL of 1× phosphate buffered saline (PBS) solution, resuspended in 35 µL of 50% glycerol solution, mounted on a microscope slide, and imaged using a Leica DM 750 microscope as described above for the cytological analysis of anthers.

## Supplementary Material

Appendix 01 (PDF)

Dataset S01 (XLSX)

Dataset S02 (XLSX)

Dataset S03 (XLSX)

Dataset S04 (XLSX)

Dataset S05 (XLSX)

Dataset S06 (XLSX)

Dataset S07 (XLSX)

Dataset S08 (XLSX)

Dataset S09 (XLSX)

Dataset S10 (XLSX)

Dataset S11 (XLSX)

Dataset S12 (XLSX)

Dataset S13 (XLSX)

Dataset S14 (XLSX)

Dataset S15 (XLSX)

Dataset S16 (XLSX)

Dataset S17 (XLSX)

Dataset S18 (XLSX)

## Data Availability

The high-throughput sequencing data reported in this article have been deposited in the NCBI Gene Expression Omnibus (53) and are accessible through GEO Series accession number GSE254584.
